# Early Drug Discovery and Development of Novel Cancer Therapeutics Targeting DNA Polymerase Eta (POLH)

**DOI:** 10.3389/fonc.2021.778925

**Published:** 2021-11-19

**Authors:** David M. Wilson, Matthew A. J. Duncton, Caleb Chang, Christie Lee Luo, Taxiarchis M. Georgiadis, Patricia Pellicena, Ashley M. Deacon, Yang Gao, Debanu Das

**Affiliations:** ^1^ XPose Therapeutics, Inc., San Carlos, CA, United States; ^2^ Biomedical Research Institute, Hasselt University, Diepenbeek, Belgium & Boost Scientific, Heusden-Zolder, Belgium; ^3^ Department of BioSciences, Rice University, Houston, TX, United States

**Keywords:** fragment-based drug discovery (FBDD), structure-based drug discovery (SBDD), X-ray crystallography, cancer therapeutics, DNA damage response (DDR), polymerases, Pol eta, POLH

## Abstract

Polymerase eta (or Pol η or POLH) is a specialized DNA polymerase that is able to bypass certain blocking lesions, such as those generated by ultraviolet radiation (UVR) or cisplatin, and is deployed to replication foci for translesion synthesis as part of the DNA damage response (DDR). Inherited defects in the gene encoding POLH (a.k.a., XPV) are associated with the rare, sun-sensitive, cancer-prone disorder, xeroderma pigmentosum, owing to the enzyme’s ability to accurately bypass UVR-induced thymine dimers. In standard-of-care cancer therapies involving platinum-based clinical agents, e.g., cisplatin or oxaliplatin, POLH can bypass platinum-DNA adducts, negating benefits of the treatment and enabling drug resistance. POLH inhibition can sensitize cells to platinum-based chemotherapies, and the polymerase has also been implicated in resistance to nucleoside analogs, such as gemcitabine. POLH overexpression has been linked to the development of chemoresistance in several cancers, including lung, ovarian, and bladder. Co-inhibition of POLH and the ATR serine/threonine kinase, another DDR protein, causes synthetic lethality in a range of cancers, reinforcing that POLH is an emerging target for the development of novel oncology therapeutics. Using a fragment-based drug discovery approach in combination with an optimized crystallization screen, we have solved the first X-ray crystal structures of small novel drug-like compounds, i.e., fragments, bound to POLH, as starting points for the design of POLH inhibitors. The intrinsic molecular resolution afforded by the method can be quickly exploited in fragment growth and elaboration as well as analog scoping and scaffold hopping using medicinal and computational chemistry to advance hits to lead. An initial small round of medicinal chemistry has resulted in inhibitors with a range of functional activity in an *in vitro* biochemical assay, leading to the rapid identification of an inhibitor to advance to subsequent rounds of chemistry to generate a lead compound. Importantly, our chemical matter is different from the traditional nucleoside analog-based approaches for targeting DNA polymerases.

## Introduction

Cancer will directly affect the lives of over one-third of the population, with the process of carcinogenesis involving (at least) six biological phenomenon/hallmarks (1): sustaining proliferative signaling, evading growth suppressors, resisting cell death, enabling replicative immortality, inducing angiogenesis, and activating invasion and metastasis. Many of these hallmarks, if not all, can be fostered by genomic instability that arises due to excessive DNA damage or defects in DNA damage response (DDR) components. The upregulation of certain DDR pathways is also a compensatory mechanism employed by cancer cells to adapt to the elevated background levels of DNA damage imparted by their rapid cell division and increased metabolism (2) or to survive treatment-related DNA-damaging agents, like certain forms of chemotherapy and radiotherapy (3, 4). The recognition that these intrinsic changes in the DDR (i.e., sporadic inactivation or upregulation) offer therapeutic opportunities has led to advances in cancer treatment efficacy. Most notably, the discovery that homologous recombination repair (HRR)-defective breast and ovarian cancers are uniquely sensitive to poly (ADP-ribose) polymerase (PARP) inhibitors *via* a mechanism broadly referred to as synthetic lethality (SL) has led to improved drug design/application and better outcomes for many of these cancer-affected individuals (5). Thus, further development of DDR inhibitors to combat both intrinsic and acquired drug resistance presents an enormous therapeutic opportunity that could widen the repertoire of initial treatment options and re-sensitize cells to therapies that have failed due to upregulation of DDR pathways. Two primary therapeutic approaches involving DDR targeting could include: combinatorial treatments that involve anticancer genotoxic agents and SL, a phenomenon that as mentioned above exploits a sporadic DDR defect to achieve cancer-specific cell death. Here, we provide results on our early drug discovery efforts around the identification and development of novel inhibitors targeting human DNA polymerase eta (Pol η or POLH).

DDR is an intricate system involving damage recognition, cell cycle regulation, DNA repair, and cell fate determination, playing a significant role in cancer etiology and therapy. POLH, a.k.a., xeroderma pigmentosum variant (XPV) protein, is a translesion DNA polymerase that is a member of the Y family of polymerases (6, 7). The enzyme exhibits low fidelity on undamaged DNA, yet accurately copies ultraviolet (UV) light-induced dithymine cyclobutane pyrimidine dimers (CPDs) by inserting A-A opposite the lesion. In addition to UV-induced DNA damage, POLH has been shown to bypass cisplatin adducts, as well as oxaliplatin adducts (8–14). Additional studies suggest that POLH may also play an important role in oxidative stress resistance, likely by carrying out translesion synthesis (TLS) (15, 16) of bulky oxidative base lesions, such as cyclopurines (17–19).

Consistent with the known biochemistry, elevated POLH expression correlates with reduced cisplatin sensitivity in models of lung and bladder cancer (8). Strategic downregulation of POLH in these cases re-sensitizes cancer cells to cisplatin treatment, supporting targeting of the polymerase in certain situations of acquired drug resistance. Suppression of POLH expression also enhances cisplatin-induced apoptosis of cancer stem cells isolated from both ovarian cancer cell lines and primary tumors (10). Furthermore, studies indicate that POLH is a predictive factor of treatment response and survival of metastatic gastric adenocarcinoma patients receiving oxaliplatin-based first-line chemotherapy (20). In addition to its well-established role in platin drug resistance, preclinical studies indicate that POLH-deficient cells are 3-fold more sensitive to the nucleoside analogs β-D-arabinofuranosylcytosine and gemcitabine, and even more sensitive (10-fold) to gemcitabine/cisplatin combination treatment (21), a commonly used clinical regimen for treating a wide spectrum of cancers, including bladder, pancreatic, ovarian, cervical, and non-small cell lung. Additional investigations have revealed that co-inhibition of POLH and ATR, a protein central to the replicative stress response, offers a SL approach for the treatment of a range of cancer types (22, 23). Notably, ATR inhibitors are progressing well in the clinic (24, 25), and ATR haploinsufficiency, arising due to somatic mutations in one allele, is frequent in certain cancers (26), presenting therapeutic opportunities for POLH inhibition. Despite the promise of targeting POLH in anticancer therapies, clinical inhibitors have yet to be developed.

It is worth emphasizing that polymerases are validated targets in several clinical paradigms. For example, one of the most important polymerases against which medicines have been made is the DNA polymerase of HIV-1 (i.e., the reverse transcriptase, RT), the main target of antiretroviral therapies involving both nucleotide and non-nucleotide inhibitors (NRTIs and NNRTIs). In this context, it is intriguing that POLH has also been recently found to be a human RT, although the precise biological role of this biochemical function is still unclear (27). Other examples include the development of inhibitors against viral RNA polymerases (RdRp), such as the drug remdesivir (28), which was first developed as an Ebola Virus RdRp inhibitor (29) and is now being pursued in SARS-CoV-2 (30), as well as the clinically-approved anti-Hepatitis C NSB5 polymerase drug sofosbuvir (31). In addition to PARP (see above), POLQ (DNA polymerase theta), an enzyme involved in double strand break repair, is another DDR polymerase of current interest in the design of new oncology therapeutics (32), including in a SL paradigm involving BRCA1/2 mutations.

With the value in targeting DNA polymerases in general and POLH in particular, specifically in the context of new oncology therapeutics, it is not surprising that some attempts have been made in this direction. Previous work on developing POLH inhibitors focused on compounds derived from N-aryl-substituted indole barbituric acid (IBA), indole thiobarbituric acid (ITBA), and indole quinuclidine scaffolds (9, 33), which are predicted to interfere with template DNA orientation. However, these compounds have yet to advance further, and our assessment based on information available is that could be due to: (i) precise target engagement/hit validation is unknown due to absence of crystal structures, preventing further interaction-based optimization, and/or (ii) suitability of these compounds for further chemistry tractability/optimization.

To overcome the bottleneck of lack of information regarding target engagement of an identified inhibitor, our approach integrates ABS-OneStep (Accelero Biostructures, CA), a fragment-based drug discovery (FBDD) approach that uses high-throughput X-ray crystallographic screening of small molecule fragment libraries for hit generation (34). This strategy, coupled with iterative structure-guided optimization/structure-based drug discovery (SBDD), facilitates the rational development of novel therapeutics, namely small molecule inhibitors or target ligands in a targeted protein degradation approach involving a proteolysis-targeting chimera. Here, we report on the first high resolution crystal structures of POLH bound to distinct fragments that reveal direct target engagement, binding site, binding pose and protein-ligand interactions; and describe functional activity of our hits.

## Materials and Methods

### Protein Expression and Purification

Wild-type human POLH (residues 1–432) was cloned into a modified pET28p vector with a N-terminal 6-histidine tag and a PreScission Protease cleavage site. For protein expression, this POLH plasmid was transformed into BL21 DE3 *E. coli* cells. When the optical density of the *E. coli* cells reached 0.8, isopropyl ß-D-1-thiogalactopyranoside (IPTG) was added to a final concentration of 1 µM IPTG. After 20 hrs (16°C) of induction, the cell paste was collected *via* centrifugation and resuspended in a buffer that contained 20 mM Tris (pH 7.5), 1 M NaCl, 20 mM imidazole, and 5 mM ß-mercaptoethanol (BME). After sonication, POLH was loaded onto a HisTrap HP column (GE Healthcare), which was pre-equilibrated with a buffer that contained 20 mM Tris (pH 7.5), 1 M NaCl, 20 mM imidazole, and 5 mM BME. The column was washed with 300 mL of buffer to remove nonspecific bound proteins and was eluted with buffer that contained 20 mM Tris (pH 7.5), 1 M NaCl, 300 mM imidazole, and 3 mM dithiothreitol (DTT). The eluted POLH was incubated with PreScission Protease to cleave the N-terminal 6-histidine-tag. Subsequently, POLH was buffer-exchanged and desalted to 20 mM 2-(N-morpholino)ethanesulfonic acid (MES) (pH 6.0), 250 mM KCl, 10% glycerol, 0.1 mM ethylenediaminetetraacetic acid (EDTA), and 3 mM DTT and was loaded onto a MonoS 10/100 column (GE Healthcare). Protein was eluted with an increasing salt (KCl) gradient. Finally, POLH was purified with a Superdex 200 10/300 GL column (GE Healthcare) with a buffer that contained 20 mM Tris (pH 7.5), 450 mM KCl, and 3 mM DTT.

### Hit Generation by High-Throughput X-Ray Crystallography-Based Screening of Fragment Library

Hit generation by screening a diverse fragment library and the crystal structures of their binding sites in a single step was performed by using the ABS-OneStep platform (Accelero Biostructures, CA) (34). Briefly, crystals of the apo binary POLH-DNA complex were reproduced (18) and the crystallization optimized to generate several hundred crystals of relatively uniform quality for library screening directly by ultra-high throughput X-ray crystallography. Approximately 300 crystals of the POLH-DNA complex were then used to screen the ABS-Real300 (Accelero Biostructures) 300-fragment library, one fragment at a time. A total of approximately 300 individual X-ray diffraction data sets were collected at SSRL on beamline 9-2 using the BLU-ICE (35) data collection environment. The data sets were collected at 100 K, using a Pilatus 6M detector (Dectris). The data were processed with data processing and structure determination pipelines within the ABS-OneStep platform using XDS (36) and CCP4 (37), with structure determination performed by molecular replacement using our 1.5Å resolution apo POLH-DNA binary complex as the search template.

### DNA Synthesis Assay for Screening Inhibitors

POLH biochemical assays testing nucleotide incorporation activity were performed as previously described (38). The reaction mixture contained 3 nM POLH, 200 nM DNA, 50 µM dNTP, 150 mM KCl, 45 mM Tris (pH 7.5), 5 mM MgCl_2_, 10 mM DTT, 0.1 mg/mL bovine serum albumin, 5% glycerol, and 10% DMSO, and 0.01-20 mM inhibitory compound. Initial tests and next phase assays were executed using DNA template (5’-GAG TCA TGT TTA CGC TAG GCA C-3’) and 5’-fluorescein- labeled primer (5’-GTGCCTAGCGTAA-3’). Reactions were conducted at 37°C for 5 min and were stopped by adding formamide quench buffer to the final concentrations of 40% formamide, 50 mM EDTA (pH 8.0), 0.1 mg/ml xylene cyanol, and 0.1 mg/ml bromophenol. After heating to 97°C for 5 min and immediately placing it on ice, reaction products were resolved on 22.5% polyacrylamide urea gels. The gels were visualized by a Sapphire Biomolecular Imager and quantified using the built-in software. Visual representation of the acquired data was rendered in Graph Prism. For the initial inhibitor tests, each compound was assayed for any inhibitory effect on POLH nucleotide incorporation activity at different concentrations (0.01, 0.1, and 1 mM for the first batch and 0.2, 2, and 20 mM for a second batch). The gels were visualized and quantified by a Sapphire Biomolecular Imager using the built-in software.

For the compounds that exhibited signs of inhibition, each compound was serially diluted and added to a reaction mixture to a final concentration of 0.01- 20 mM. The reaction mixture contained 3 nM POLH, 200 nM DNA, 50 µM dATP, 150 mM KCl, 45 mM Tris (pH 7.5), 5 mM MgCl_2_, 10 mM DTT, 0.1 mg/mL bovine serum albumin, 5% glycerol, and 10% DMSO. Assays were performed and examined similarly as in the initial test. Quantification of IC50 and fitting was executed by Graph Prism.

## Results

### Determination of Apo POLH-DNA Binary Complex Crystal Structure

**Figure 1:**
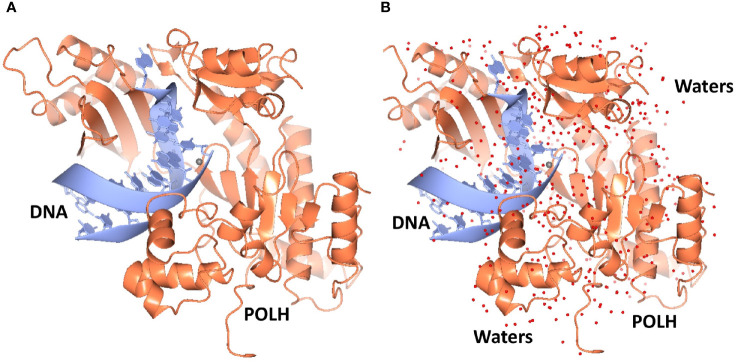
**(A)** Ribbon representation of the highest resolution 1.5Å crystal structure of the POLH-DNA binary complex. **(B)** Determination of water structure (red spheres) revealing details of water-mediated interactions, to aid structure-based drug discovery efforts on POLH.

**Figure 2:**
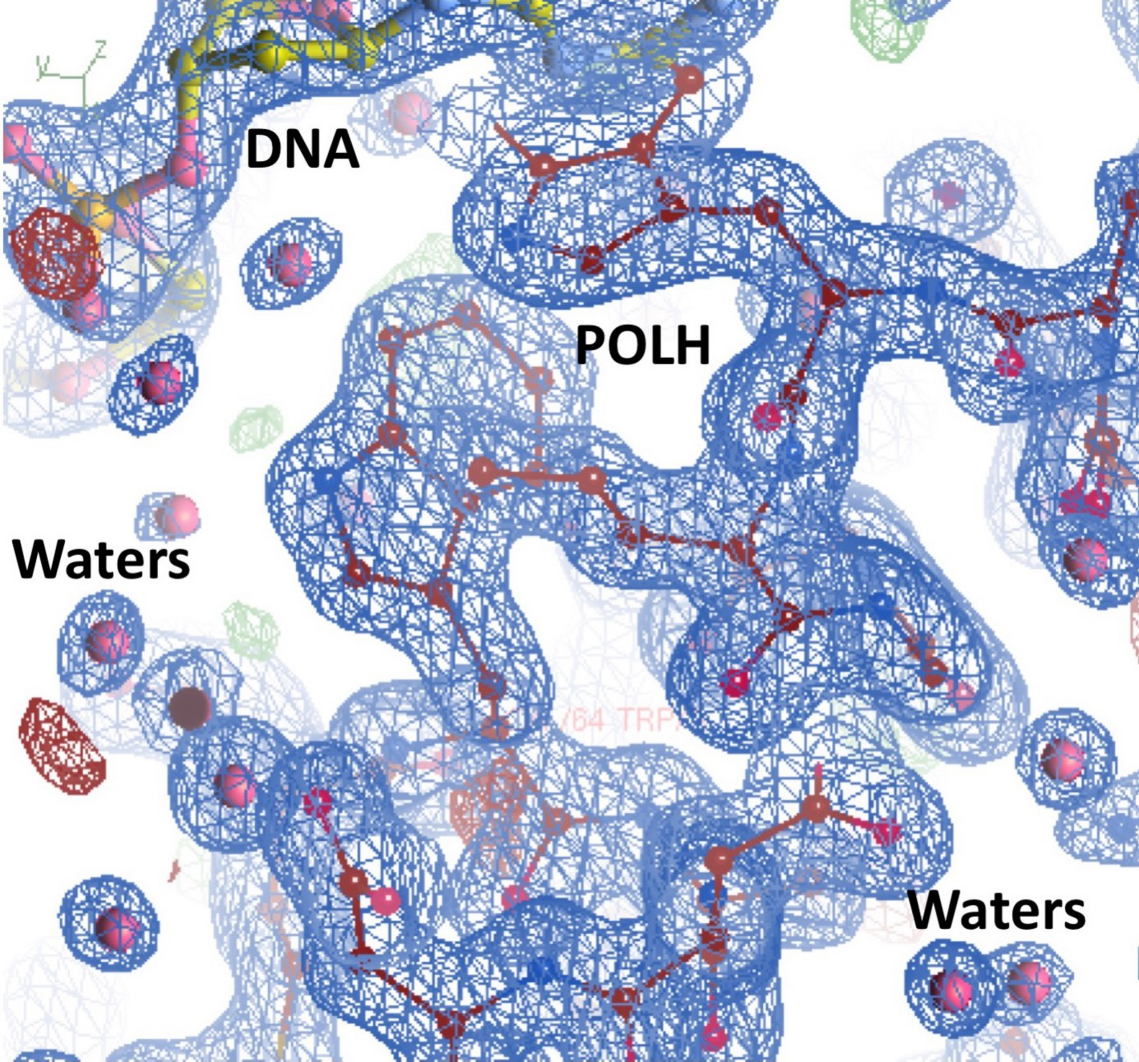
Highest resolution 1.5Å crystal structure of the POLH-DNA binary complex with R/R_free_ 13.0/19.0% in a 2fo-fc electron density map at 1.0σ map contour, revealing details of water-mediated interactions, in aid of our structure-based drug discovery efforts on POLH.

Crystals of the apo binary POLH-DNA complex were reproduced (18), and we generated several hundred crystals of relatively uniform quality for library screening directly by ultra-high throughput X-ray crystallography (see below). During the optimization process, we obtained the highest resolution crystal structure of a POLH-DNA binary complex to date, at 1.5Å resolution, which was refined to a crystallographic R/R_free_ of 13.0/19.0% (**Figure 1**). This structure revealed details of water-mediated interactions in the binary complex that we can utilize for our structure-guided inhibitor optimization (**Figure 2**), and provided us with a very high resolution binary complex structure to use as our template for crystal structure determination by molecular replacement of fragment-bound crystal structures.

### Fragment Hit Generation and Hit Elaboration for Hit-to-Lead Development

**Figure 3:**
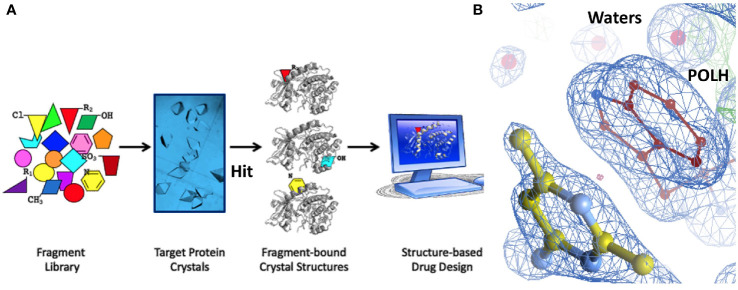
**(A)** Schematic of our approach to hit generation by screening fragment libraries directly by X-ray crystallography in a primary screen. **(B)** 1.7Å crystal structure of a POLH-DNA-Hit ternary complex with R/R_free_ 16.7/20.7% in a 2fo-fc electron density map at 1. 5σ map contour.

Hit identification was achieved in a single step using ABS-OneStep, which combines fragment-based screening with X-ray crystallography. Using approximately 300 crystals of the POLH-DNA binary complex and screening a diverse, unbiased, 300-fragment library, one fragment at a time, produced four hits, resulting in a hit rate of 1.3%. A total of approximately 300 individual X-ray diffraction data sets were collected, processed, and crystal structures determined. All crystallographic data sets were approximately in the ~1.7-2.2 Å resolution range with reasonable crystallographic R/R_free_ values. A screening schematic for hit generation and a representative hit (XPTx-0267) from a 1.7 Å crystal structure is shown in a partial view interacting with POLH (**Figure 3**). Due to intellectual property considerations, high resolution details of compound engagement with POLH or specifics of the fragment growth cannot be shown at this time.

### Inhibitor Validation by Biochemical Assays

**Figure 4:**
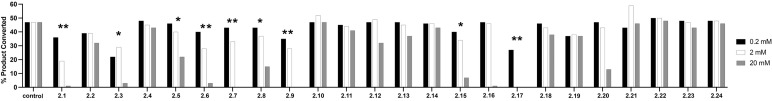
Initial pass at detecting functional activity in an *in vitro* assay for DNA synthesis. ** Represents compounds that had a 20% drop in product conversion at both 20 mM and 2 mM, * represents compounds that had a 20% drop in product conversion at only 20 mM.

**Figure 5:**
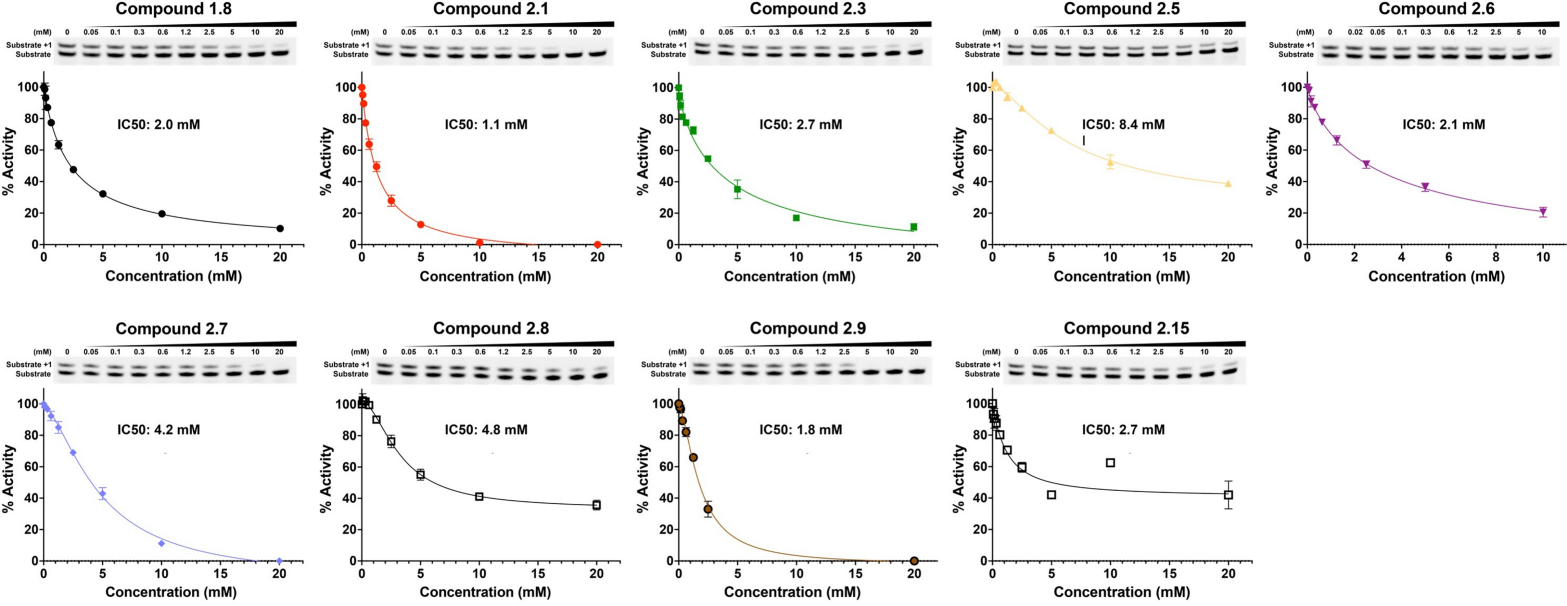
IC50 determination for some representative compounds an *in vitro* assay for DNA synthesis.

**Figure 6:**
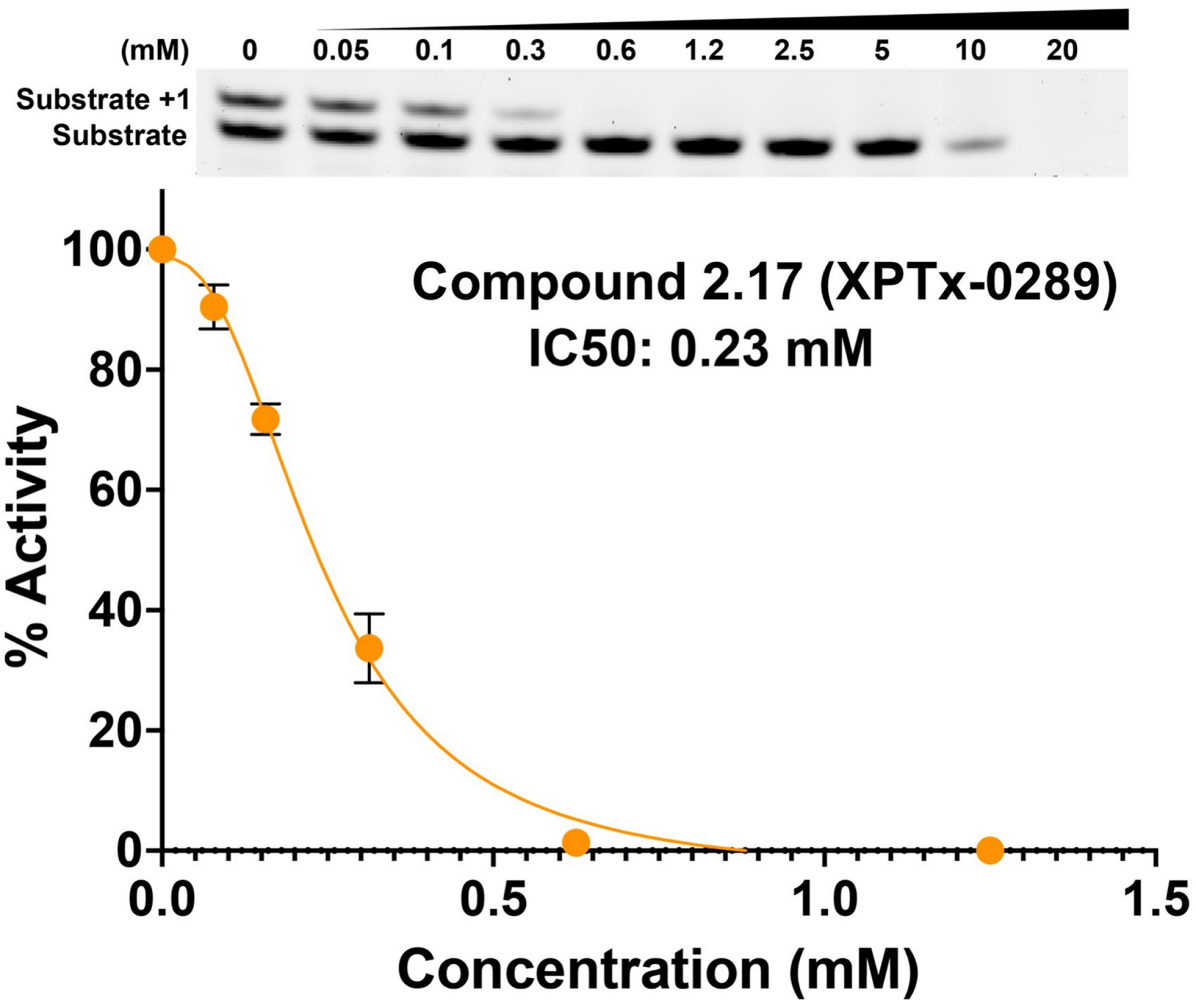
Concentration-dependent inhibition of DNA synthesis by compound XPTx-0289.

Fragment hits from the screen were subjected to fragment growth strategies, such as alternating the functional groups, analog growth, and scaffold hopping, by our in-house medicinal chemistry team. An initial limited iteration of fragment elaboration led to the selection of 40 compounds for testing in an *in vitro* nucleotide incorporation (POLH) biochemical assay as previously described (38). The assay was performed in two steps: an initial pass at detecting functional activity at either 0.01, 0.1, and 1 mM of the compound; or 0.2, 2, and 20 mM for a second batch of the compounds (**Figure 4**), followed by a more detailed pass at different compound concentrations to determine IC50 and Hill slopes. About 15 of the 40 compounds subjected to the first step were advanced to the second step for detailed measurements (**Figure 5**). In these follow-up studies, we obtained one compound with a submillimolar IC50 (230 µM), about eight compounds with IC50 ~1-5 mM, and one compound with an IC50 of ~8 mM; all had Hill slopes of ~0.8-2.4. Having in hand a set of compounds displaying varied inhibition levels provides alternative starting points and/or development paths. Based on the initial profiling, our approach quickly led to the identification of our lead compound, XPTx-0289, with an IC50 of 230 µM (**Figure 6**), with additional backup compounds also being identified.

## Discussion

FBDD holds immense promise in the development of target-specific novel and active chemical matter, as demonstrated by the advancement of several medicines to the clinic. Our FBDD strategy has quickly produced functional compounds from weak hits identified in our initial library screens, irrespective of where the compounds were on the potency spectrum from weak to strong. Indeed, we have shown for two of our targets, POLH (herein) and apurinic/apyrimidinic endonuclease 1 (APE1; see more below), where biochemical assays could not detect functional activity of the original fragments, that from a single/initial round of fragment growth and expansion, we can rapidly facilitate hit-to-lead conversion using just the empirical knowledge intrinsic to the crystal structures. While library screening by biophysical assays like SPR (Surface Plasmon Resonance) and NMR (Nuclear Magnetic Resonance) are better than biochemical assays at detecting protein-fragment interactions during library screening, they do not provide information on binding site, binding pose, or protein-ligand interactions. Biophysical assays also do not separate hits into orthosteric or allosteric site binders or reveal potentially new binding hotspots. By integrating a method with the widest detection range (i.e., X-ray crystallography), the FBDD approach allows one not to miss relevant chemical matter during screening and facilitates rapid hit-to-lead optimization efforts *via* a structure-guided approach.

While the measured potencies for XPTx-0289 (IC50 230 µM) and XPTx-0267 (2 mM) may appear low, such values, and even weaker, are typical for starting hits in FBDD projects. For instance, recent examples of programs successfully advancing fragments with initial low potencies (>2 mM Kd or IC50) include inhibitors against Cyclophilin D (39), Mycobacterium tuberculosis InhA (40), and WDR5-Myc (41). For our DDR target APE1, we now have in hand a lead inhibitor with a Ki of 350 nM (IC50 ~500 nM) after a single round of fragment expansions encompassing ~200 compounds based on the starting hit from a similar crystallography-based primary screen using ABS-OneStep (34). Notably, in our APE1 effort, the original fragment hit had undetectable activity as an inhibitor of APE1 AP site cleavage activity in a standard biochemical assay. The rapid advancement of an initial hit to significantly improved congener inhibitors demonstrates the power of our platform to rapidly execute hit-to-lead campaigns for the development of target-specific inhibitors. Indeed, XPTx-0289 is now ready to advance to lead generation in a hit-to-lead campaign, in conjunction with cellular TLS and co-inhibition assays.

## Data Availability Statement

The datasets presented in this article are not readily available because of intellectual property considerations. Requests to access the datasets should be directed to info@xposetx.com.

## Author Contributions

DD and AD contributed POLH-DNA binary complex crystallization, library screening for hit generation and X-ray crystallography, and structure analyses. MD and TG contributed to hit expansions and fragment elaborations. CC, CL, and YG contributed to POLH protein and binary complex production, and biochemical assays. DD, AD, MD, PP, DW, TG, CC, and YG contributed to the interpretation of results and critical review of the manuscript. DW, DD, CC, and YG contributed to writing the manuscript. All authors contributed to the article and approved the submitted version.

## Funding

Research included in this publication was supported by the National Center For Advancing Translational Sciences of the NIH under Award Number R43 TR001736, and the National Institute of General Medical Sciences of the NIH under Award Number R44 GM132796, to Accelero Biostructures Inc.; National Cancer Institute of the NIH under Award Number R43 CA254552 to XPose Therapeutics, Inc.; and Cancer Prevention & Research Institute of Texas (CPRIT) Award RR190046 and Welch Foundation Grant Number C-2033-20200401 to YG. CC was supported by a fellowship from the Houston Area Molecular Biophysics Program (NIH Grant No. T32 GM008280, Program Director Dr. Theodore Wensel).

## Conflict of Interest

Authors DW, MD, TG, PP, AD and DD receive compensation, including stock-based awards from XPose Therapeutics, Inc.

The remaining authors declare that the research was conducted in the absence of any commercial or financial relationships that could be construed as a potential conflict of interest.

The authors declare that this study received funding from XPose Therapeutics, Inc. and Accelero Biostructures, Inc. The funders had the following involvement in the study: study design, data collection and analysis, decision to publish, or preparation of the manuscript.

## Publisher’s Note

All claims expressed in this article are solely those of the authors and do not necessarily represent those of their affiliated organizations, or those of the publisher, the editors and the reviewers. Any product that may be evaluated in this article, or claim that may be made by its manufacturer, is not guaranteed or endorsed by the publisher.
